# Agreement between self-reported healthcare service use and administrative records in a longitudinal study of adults recently released from prison

**DOI:** 10.1186/s40352-016-0042-x

**Published:** 2016-11-23

**Authors:** Megan Carroll, Georgina Sutherland, Anna Kemp-Casey, Stuart A. Kinner

**Affiliations:** 1 Melbourne School of Population and Global Health, University of Melbourne, Melbourne, Australia; 2 Centre for Health Services Research, School of Population Health, University of Western Australia, Perth, Australia; 3 Griffith Criminology Institute & Menzies Health Institute Queensland, Griffith University, Brisbane, Australia; 4 Mater Research Institute-UQ, University of Queensland, Brisbane, Australia

**Keywords:** Self report, Data linkage, Data quality, Prisoners, Ex-prisoner

## Abstract

**Background:**

Studies of healthcare service use often rely on self-reported data, especially in disadvantaged populations. Despite this, the reliability of self-reported healthcare service use is often questioned and routinely-collected, administrative data are usually considered preferable. In this paper we examine the agreement between self-reported healthcare service use and administrative records, in a large cohort of adults recently released from prison in Australia.

**Methods:**

Baseline interviews within 6 weeks of expected release from prison and follow-up interviews at 1, 3 and 6 months post-release were linked to routinely-collected, administrative health records over the same time period. Outcomes of interest included use of primary care, emergency department presentation, hospitalisation and dispensing of subsidised pharmaceuticals. Kappa statistics and positive and negative predictive values were calculated for each service type and time point, and a modified Poisson regression was used to identify participant characteristics associated with better agreement.

**Results:**

864 participants completed interviews and were successfully linked to administrative records. There was good agreement between self-report and administrative health records. Agreement between data sources at 1 month was best for psychotropic medications (kappa = 0.79) and primary care visits (kappa = 0.69).

**Conclusion:**

Despite a common perception that studies using self-reported data are subject to bias, particularly among the disadvantaged, our findings suggest that self-reported healthcare may be valid in vulnerable populations.

## Background

Ascertaining how, when and how often consumers use healthcare services is critical to effective healthcare policy and funding. Self-report is a common method of collecting information on the way people use healthcare services (Bhandari and Wagner 2006). As such, determining the accuracy of self-reported, health-related data has important implications for future delivery and accessibility of healthcare services, because the utility of these data depends on their accuracy. A key advantage of self-reported data is that they can be systematically collected for a large sample at relatively low cost and for some populations, there may be no other sources of data available.

Given its frequent use in research, many studies have attempted to determine the validity of self-reported healthcare service utilisation (Bhandari and Wagner, 2006; Ritter et al. 2001). Accuracy of self-report has been shown to be influenced by the ability and willingness of respondents to recall events accurately, and can be affected by time since the event, salience of the event, frequency of events and various population-specific characteristics such as education and age (Gelberg and Siecke 1997, Reijneveld and Stronks 2001, Raina et al. 2002, Bhandari and Wagner 2006, Glass and Bucholz 2011). Despite this existing literature, there is often doubt about the accuracy of self-report among vulnerable groups such as drug users and ex-prisoners, although there is little evidence that self-report to support this (Gelberg and Siecke 1997, Reijneveld and Stronks 2001, Raina et al. 2002, Glass and Bucholz 2011 (Somers et al., 2016). This doubt can lead to a distrust of research based on self-report with these populations, and may contribute to suspicion of patients’ self-reported medical histories.

Recently released prisoners are vulnerable to multiple types of social exclusion and disadvantage, and are at high risk of poor health outcomes including drug overdose, injury, blood-borne viral infection and avoidable mortality (Kinner and Wang 2014). In Australia, universal health insurance coverage ensures that all ex-prisoners have access to healthcare and as such, service use in this population is reasonably well documented in routinely-collected data. However in some other countries, including the United States, the majority of ex-prisoners do not have health insurance (Morrissey et al. 2006), such that neither health outcomes nor patterns of healthcare use after release from prison are well understood. Those studies that do use routinely-collected, administrative data are often not representative of the wider ex-prisoner population; for example only including prisoners eligible for particular government benefit schemes (Wang et al. 2013), only those living with HIV, or only those attending particular hospitals (Frank et al. 2013). Partly for this reason, many studies of health outcomes and healthcare use in ex-prisoners rely on self-report, often noting that this is a potential limitation to the accuracy of their findings (Lorvick et al. 2015).

Australia’s healthcare system, available to all residents, is largely funded by the federal government and administered by a combination of state and federal programs. The nature of this system is such that administrative data regarding use of healthcare services, including the dispensing of prescription medications, are collected at an individual level for all residents. While these data are collected primarily for funding and administrative purposes, they can be used as an objective measure of healthcare service use, allowing for a comparison with self-reported data that is not possible in many settings.

Using a combination of self-reported and routinely-collected data from a large, representative sample of ex-prisoners in Australia, we aimed to assess the validity of self-reported healthcare and medication use in the first six months after release from custody. In addition, we sought to identify individual characteristics associated with higher levels of agreement between self-reported and administrative data.

## Methods

### Setting

This study compares the self-reported healthcare use of adults released from prison in Queensland, Australia, with administrative health records in the 6 months following release from custody. Participants were recruited for baseline interviews within 6 weeks of expected release from custody and completed follow-up interviews approximately 1, 3 and 6 months after release. Administrative data reflecting use of primary care, emergency department presentation, hospitalisation and dispensing of subsidised pharmaceuticals over the same time period were obtained via probabilistic data linkage. Ethics approval for the study was granted by the University of Queensland Behavioural and Social Sciences Ethical Review Committee. Approval for data linkage was also granted by the Commonwealth Department of Human Services and the Queensland Department of Health under the *Public Health Act 2005*.

### Measures

Baseline interviews covered a range of demographic information including age, sex, Indigenous status (Aboriginal or Torres Strait Islander), education, employment history, substance use history, mental and physical health status. Alcohol dependence in the year before entering prison was assessed using the Alcohol Use Disorders Identification Test (AUDIT), and those scoring 20 or higher were considered possibly dependent (Babor et al. 1992). The Hayes Ability Screening Index (HASI) was used to assess participants for possible intellectual disability (Hayes 2002). In order to increase specificity of this measure (Dias et al. 2013), those who scored <85 on the HASI and reported a diagnosis of intellectual disability and/or attendance at a special school were considered to have intellectual disability. With participant consent, prison medical records were accessed to identify current medications and the results of tests for hepatitis B and C, HIV and tuberculosis. Medications were classified into treatment categories according to the MIMS classification system (MIMS Australia Pty Ltd)., with a binary variable created to indicate whether participants were receiving central nervous system medications (predominantly psychotropic) at the time of interview.

### Self-reported healthcare use

Follow-up interviews assessed three different types of healthcare service use: consultations with primary care physicians (known as general practitioners or GPs in Australia), hospital admissions and emergency department (ED) presentations. For contact with GPs, participants were asked “How many times since your release from custody/last interview did you see a GP?” Responses were dichotomised into ‘none’ versus ‘1 or more times’. For hospital admissions, participants were asked “Since your release from prison/last interview, have you been hospitalised or admitted to an in-patient facility for any reason (e.g., physical health, mental health, drug treatment)?” (yes/no). For ED presentation, participants were asked “Apart from a GP, which services have you contacted about your general health (e.g., emergency department; ED) since your release from custody?” and free text responses were post-coded to create a binary ED presentation variable.

Participants also reported whether they were currently taking any medications (yes/no) and, if yes, the name, dosage and frequency of administration. The reported medication names were then coded according to the Anatomical Therapeutic Chemical (ATC) classification system (WHO Collaborating Centre for Drug Statistics Methodology 2014). Medication names that were unclear or nonsensical were coded to missing. Medication types were defined based on their ATC pharmacological subgroup. Dichotomous variables were created to indicate whether participants reported taking, 1) any medication and 2) three common types of medication (antidepressants, antipsychotics and lipid-modifiers). We selected these three medication types based on their common usage and clinical relevance in this population.

### Administrative data

Objective healthcare utilisation data were obtained by probabilistic linkage, using participant names (including all aliases), birthdates and last known locations, with state and federal administrative databases. Data on hospital admissions and emergency department presentations were provided by Queensland Health, with linkage undertaken by the Queensland Health Data Linkage Unit. As Australian hospitals are under state jurisdiction, these records cover hospitals and emergency departments throughout Queensland.

Records of GP contacts were obtained from Medicare Benefits Schedule (MBS) claims data (http://www.health.gov.au/internet/mbsonline/publishing.nsf/Content/Medicare-Benefits-Schedule-MBS-1). The MBS is a national, universal healthcare scheme that subsidises GP visits and other outpatient services such as optometry. For the purposes of this paper, GP contact was defined as all GP attendance items as well as specialty items (e.g., the Indigenous health check) that are performed by a GP or another professional, such as a practice nurse, on behalf of a GP.

Medication data were sourced from Pharmaceutical Benefits Scheme (PBS) claims (http://www.pbs.gov.au/info/about-the-pbs). The PBS is a national scheme to subsidise prescription medications for all Australians. Low income earners and those receiving unemployment or other government pension benefits pay a ‘concessional’ co-payment which is much lower than the ‘general’ population co-payment. Participants were considered to be currently taking medications if they had filled a prescription within “number of days supplied” ±5 days of interview (e.g., 28-day prescription would be filled between 33 days before and 5 days after interview.) As with self-reported medications, we constructed dichotomous variables indicating whether participants were taking any antidepressants, antipsychotics or lipid-modifiers according to PBS records.

### Data analysis

First, we compared dichotomous self-reported healthcare use and use of medications since release/last interview with the relevant administrative records over the same time period for each participant. Levels of agreement were assessed using Cohen’s kappa statistic. Positive predictive value (PPV) and negative predictive value (NPV) were determined for each service or medication type and time point, treating administrative records as the gold standard for calculation purposes. Self-report and administrative records were deemed to be ‘concordant’ when both indicated healthcare service or medication use, or both indicated no healthcare service or medication use. Conversely, records were deemed to be ‘discordant’ when one data source indicated healthcare service or medication use, and one did not.

We used a Bland-Altman plot (Bland and Altman 1986) to examine agreement between number of self-reported GP visits and the number of visits recorded in MBS data, with responses from all three follow-up interviews included in one plot. This method was chosen as it does not rely on the assumption that either data source is a ‘gold standard’ (Bland and Altman 1986).

The proportion of participants with agreement between self-reported and MBS-recorded GP contact within 1 month of release was calculated for subgroups, based on characteristics that may be associated with poor self-report. Variables were chosen either due to previously shown association with self-report (eg education) or as potential explanations for poor self-report among this specific population (e.g., history of mental illness and drug use). As many of these characteristics are interdependent, we examined these relationship both univariately and in a multivariate model. As GP use was not a rare event, we used a multivariate Poisson approach to determine the incidence rate ratios (IRR) for predictors of accurate self-report. All variables were included in the multivariate model and, after adjusting for other factors, those that were not predictive were excluded, with the exception of age, sex and Indigenous status. All analyses were conducted using STATA version 13.0.

## Results

A total of 1325 participants were interviewed at baseline, with follow-up fractions of 69, 68, 72% at 1, 3 and 6 months respectively, resulting a total of 936 people participating in at least one follow-up interview with valid responses regarding use of healthcare services. Of these, 92% were also successfully linked to both state and federal healthcare records, giving a sample of 864 eligible for analysis. Approximately one quarter of participants (23%) were female and 20% identified as Indigenous (Aboriginal and/or Torres Strait Islander); the majority (61%) were aged 25 to 44 years (Table 1).

**Table 1:** Sample characteristics at baseline (*N* = 864)

	*N* (%)
Female	197 (22.8)
Indigenous	170 (19.7)
Mental illness ever	382 (44.2)
Educated 10+ years	519 (60.1)
Age 18–24 years	196 (22.7)
25–44 years	528 (61.1)
45+ years	140 (16.2)
Intellectual disability	75 (8.7)
Hepatitis C exposed^a^	127 (14.9)
Taking Central Nervous System medications^b^	256 (30.6)
Injected drugs ever	468 (54.2)
Opioid use weekly or more^d^	127 (14.7)
Methamphetamine use weekly or more^d^	217 (25.2)
Cannabis use weekly or more^d^	330 (38.2)
Possibly alcohol dependent (AUDIT > 20)^c^	227 (26.7)
Stable accommodation^d^	727 (84.1)
Employed^d^	475 (51.5)

^a^ *N* = 853 ^b^ *N* = 838 ^c^ *N* = 849 ^d^prior to incarceration

Table 2 shows the observed agreement between self-report and administrative data at 1, 3 and 6 months after release from prison. NPV for all healthcare services were good (range 67.5–99.2), with Kappas showing moderate agreement (range 0.31–0.69). PPV were good for GP use (>79%), however PPV was moderate and rarer events (ED presentation and hospital admission). There was evidence of under-reporting of ED presentation, and of poorer agreement between self-report and administrative records over longer time periods.

**Table 2:** Agreement between self-reported and routinely collected data for any attendance at health care, at 1, 3 and 6 months following release from prison

	Concordance *n* (%)		Discordance *n* (%)		Kappa	PPV %	NPV %
Self-report	No	Yes	Yes	No			
Records	No	Yes	No	Yes			
One month post release							
General Practitioner (*n* = 738)	343 (46)	280 (38)	69 (9)	46 (6)	0.69	80.2	88.2
Emergency Department (*n* = 808)	751 (93)	16 (2)	9 (1)	32 (4)	0.41	64.0	95.9
Hospital admission (*n* = 794)	753 (95)	19 (2)	16 (2)	6 (1)	0.62	76.0	99.2
Three months post release							
General Practitioner (*n* = 690)	270 (39)	276 (40)	72 (10)	72 (10)	0.58	79.3	78.9
Emergency Department (*n* = 744)	636 (85)	33 (4)	18 (2)	57 (8)	0.42	64.7	91.8
Hospital admission (*n* = 730)	655 (90)	37 (5)	13 (2)	25 (3)	0.63	74.0	96.3
Six months post release							
General Practitioner (*n* = 646)	195 (30)	299 (46)	58 (9)	94 (15)	0.52	76.1	67.5
Emergency Department (*n* = 686)	540 (79)	34 (5)	14 (2)	98 (14)	0.31	70.8	84.6
Hospital admission (*n* = 670)	577 (86)	28 (4)	28 (4)	37 (6)	0.41	50.0	94.0

**Fig. 1:**
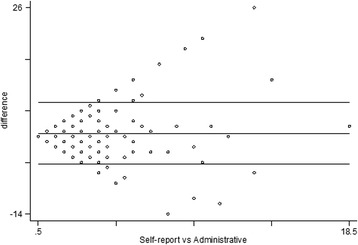
Bland-Altman plot of agreement between number of general practitioner contacts self-reported and number according to individuals administrative records for the same time period

Figure 1 is a Bland-Altman plot comparing number of GP visits by self-report with the number appearing in administrative records. The average difference between number of GP visits by self-report and MBS records increases as the total the number of visits increases, with the higher positive difference suggesting a bias towards under-reporting the true number of visits.

Table 3 shows the concordance between self-reported current medication use and prescriptions filled, both for any medication and for antidepressants, antipsychotics, and lipid-modifiers. Agreement between self-report and PBS records for current medication use was good (Kappas range 0.57–0.84), although slightly better for psychotropic medications (antidepressants range 0.68–0.79, antipsychotics range 0.59–0.84) than for lipid-modifiers (range 0.57–0.63).

**Table 3:** Agreement between self-report and PBS claims of current medication, at 1, 3 and 6 months following release from prison

	Concordance *n* (%)		Discordance *n* (%)		Kappa	PPV %	NPV %
Self-report	No	Yes	Yes	No			
Records	No	Yes	No	Yes			
One month (*N* = 747)							
Any Medication	401 (54)	211 (28)	98 (13)	37 (5)	0.62	68.3	91.6
Antidepressant	630 (84)	81 (11)	24 (3)	12 (2)	0.79	77.1	98.1
Antipsychotic	675 (90)	42 (6)	23 (3)	7 (1)	0.72	64.6	99.0
Lipid-modifier	710 (95)	17 (2)	9 (1)	11 (1)	0.62	65.4	98.5
Three months (*N* = 698)							
Any Medication	385 (55)	203 (29)	84 (12)	26 (4)	0.66	70.7	93.7
Antidepressant	573 (82)	71 (10)	45 (6)	9 (1)	0.68	61.2	98.5
Antipsychotic	626 (90)	32 (5)	27 (4)	13 (2)	0.59	54.25	98.0
Lipid-modifier	662 (95)	15 (2)	12 (2)	9 (1)	0.57	55.6	98.7
Six months (*N* = 648)							
Any Medication	334 (52)	175 (27)	84 (13)	55 (8)	0.55	67.6	85.9
Antidepressant	546 (84)	62 (10)	28 (4)	12 (2)	0.72	68.9	97.84946
Antipsychotic	586 (90)	46 (7)	10 (2)	6 (1)	0.84	82.1	99.0
Lipid-modifier	612 (94)	17 (3)	10 (2)	9 (1)	0.63	63.0	98.6

Table 4 shows the percentage of participants for whom there was agreement between any self-reported GP contact at 1 month follow-up and administrative records. No demographic or socioeconomic characteristics were associated with agreement. The strongest predictor of agreement between self-report and records was a higher number of contacts with a GP clinic (adjusted IRR = 1.14). Regular cannabis use predicted better agreement (adjusted IRR = 1.07), however agreement was lower amongst regular opioid users (adjusted IRR = 0.89).

**Table 4:** Relative risks of correctly reporting General Practitioner contact at 1 month following release from prison

		*N* (% agreed)	Unadjusted IRR (95% CI)	Adjusted IRR (95% CI)^a^
Male		571 (84.1)	-	-
Female		167 (85.6)	1. 02 (0.95–1.09)	1.02 (0.95–1.10)
Non-Indigenous		606 (84.2)	-	-
Indigenous		132 (85.6)	1.02 (0.94–1.10)	1.00 (0.93–1.09)
Age 18–24		170 (84.1)	-	-
25–44		439 (84.0)	1.00 (0.93–1.08)	0.99 (0.92–1.07)
45+		129 (85.3)	1.02 (0.93–1.13)	1.00 (0.90–1.11)
Educated < 10 years		244 (86.1)	-	-
Educated 10+ years		379 (82.2)	0.97 (0.91–1.03)	0.97 (0.91–1.03)
Unemployed^b^		338 (84.5)	-	-
Employed		400 (84.4)	1.04 (0.97–1.10)	1.05 (0.98–1.12)
Unstable accommodation^b^		111 (87.3)	-	-
Stable accommodation		627 (83.9)	0.96 (0.89–1.04)	0.96 (0.89–1.04)
No intellectual disability		675 (84.1)	-	-
Intellectual disability		63 (87.3)	1.04 (0.94–1.15)	1.04 (0.94–1.15)
No mental Illness		354 (86.2)	-	-
Mental illness diagnosed		268 (82.2)	0.95 (0.90–1.02)	0.94 (0.88–1.01)
No CNS meds		499 (84.4)	-	-
CNS meds		221 (85.5)	1.01 (0.95–1.08)	1.00 (0.94–1.08)
Not hepatitis C positive		624 (84.8)	-	-
Hepatitis C positive		87 (82.1)	0.97 (0.88–1.06)	0.97 (0.88–1.06)
Never injected drugs		296 (86.5)	-	-
Injected drugs		326 (82.5)	0.95 (0.90–1.01)	0.96 (0.89–1.02)
No alcohol dependence		539 (84.6)	-	-
Possible alcohol dependence		184 (84.2)	1.00 (0.93–1.07)	1.00 (0.93–1.08)
No regular methamphetamines^b^		560 (85.7)	-	-
Regular methamphetamines		178 (80.3)	0.94 (0.87–1.02)	0.95 (0.87–1.02)
No regular cannabis^b^		461 (83.1)	-	-
Regular cannabis		277 (86.6)	1.04 (0.98–1.11)	1.07 (1.00–1.14)*
No regular opioids^b^		627 (85.5)	-	-
Regular opioids		111 (78.4)	0.92 (0.83–1.02)	0.89 (0.80–0.98)*
Number of GP contacts^c^	0	412 (83.2)	-	-
	1	157 (79.0)*	0.95 (0.87–1.04)	0.96 (0.87–1.05)
	2+	169 (92.3)*	1.11 (1.04–1.18)	1.14 (1.06–1.21)***

*IRR* Incidence rate ratio, *CNS* Central Nervous System, *GP* General Practitioner

*P < 0.05 ***P < 0.001

^a^Adjusted for age, sex, indigenous status and number of GP visits, ^b^prior to incarceration, ^c^from MBS records

## Discussion

The aim of this study was to examine the level of agreement between self-reported and routinely-collected data on healthcare and medication use in a highly marginalised population – ex-prisoners – in the first 6 months after release from custody. We found that overall agreement between self-reported and objective healthcare use was good, with better agreement for more recent and more general information (e.g. any visit or medication). Although there was imperfect agreement between the data sources, some of this could be explained by limitations in the administrative data or in data linkage, rather than inaccurate self-report.

By using responses collected at approximately 1, 3 and 6 months following participants release from prison, we were able to compare agreement across different periods of time, with approximately 1, 2 and 3 months of time between the respective interviews. We found that agreement between self-report and administrative records was better over shorter time periods, with concordance between 7 and 11 percentage points lower when recall was over 3 months, than when it was over 1 month. This finding is consistent with other studies on recall bias, where longer time periods of recall result in less accurate reporting (Coughlin 1990). Another possible explanation for this finding relates to the way that time periods were defined in this study: the first (1 month) time period was defined as the time ‘since your release from prison’, whereas the 2 and 3 month time periods were defined as ‘since your last interview’. Release from prison is likely to be a highly salient event, which has been shown to improve the accuracy of recall (Coughlin 1990, Bhandari and Wagner 2006). Consistent with this, we observed no association between time period and reporting of medication use; these questions related to *current* medication use rather than medication use over a specified time period.

We found that agreement between self-reported medication use and administrative records was better for psychotropic than for lipid-modifying medications. This may be due to an increased salience of medicines for symptomatic conditions over those for asymptomatic conditions, with participants more aware of medications that have a more noticeable effect on daily functioning. This finding is consistent with previous research showing more accurate recall of psychotropic medications in a sample of incarcerated adults (Carroll et al. 2014). These results are of particular interest given the well-established link between serious mental illness and impaired cognitive function (McDermott and Ebmeier 2009, Aleman et al. 1999), which would suggest worse rather than better accuracy of self-report among those taking psychotropic medications.

The lower agreement for emergency department visits than GP visits may appear inconsistent with the idea that more salient events are recalled more accurately. However, the discordance was primarily due to false negatives, and may be due in part to limitations of the survey questions, rather than participant’s recall of events. Whereas participants were asked specifically the number of times they had seen a GP or been admitted to hospital, ED presentations were coded from answers to the question “Apart from a GP, what other services have you contacted about your general health?” This less direct and more ambiguous questioning may have led to variation in how participants reported ED visits.

We examined a wide range of potential correlates of agreement between self-reported GP use and GP contact according to administrative records. The variable most strongly associated with agreement was number of GP visits; those who visited a GP two or more times in the first month post-release (according to administrative records) were more likely to self-report having visited a GP at least once in this time period. This finding is likely due to it being easier to remember things that happen more often. As people who visit healthcare services more frequently tend to have poorer health, this result suggests that studies that recruit participants based on self-reported healthcare use may over-sample people with poor health, and under-sample those who attend health services less frequently.

In multivariate analyses two additional correlates of agreement between self-report and administrative records emerged. Agreement was poorer for those who reported regular opioid use before prison, but better for those who self-reported regular cannabis use before prison. While other literature has shown cognitive impairment associated with opiate use (Gruber et al. 2007), this is also seen among regular cannabis users (Schwartz et al. 1989), so does not fully explain our findings. Most markers of disadvantage that we examined were not associated with agreement. This may be due to a ‘floor effect’ for these variables but given the good agreement observed for most healthcare measures, our findings suggest that self-report can be reliable in highly disadvantaged populations.

To our knowledge this is the first study ever to examine the accuracy of self-reported healthcare use in a large cohort of ex-prisoners – a population in whom self-report is often thought to be unreliable (Harzke et al. 2006, Islam et al. 2013). The longitudinal design, large sample, and population coverage of administrative health records are key strengths. The inclusion of medication use, as well as healthcare services, provides a broader understanding of the strengths and weaknesses in self-reported healthcare use.

Our study had several limitations. One limitation is the use of state-based hospital and emergency department records, which would not have captured service use in other states. However, MBS records (which are national) include postcode of service and only 4% of claims in the study period were made interstate, suggesting that this is unlikely to have substantially biased our findings. In addition, a record linkage study of mortality in more than 42,000 ex-prisoners in Queensland (Kinner et al. 2012) found only modest attenuation of the mortality rate when state-based mortality records were used, suggesting relatively low interstate mobility in this population. A second limitation is that PBS (prescription) claims only include medications priced above the patient co-payment threshold, which would lead to under-ascertainment of low-cost medications in administrative records. However, as most participants were either low income earners or receiving government benefits, we anticipate that the majority were entitled to the ‘concessional’ co-payment, at <$6.00 AUD (purchasing power parity equivalent to $4.20 USD) (http://stats.oecd.org/Index.aspx?DataSetCode=PPP2011), such that the number of missed medications should be low. A third limitation is that MBS records may under-estimate GP use among Indigenous participants, who can access care through services operated by community-controlled Aboriginal Medical Services, which are funded through a different scheme (Queensland Aboriginal and Islander Health Council 2011). However, since Indigenous status was not a significant predictor of agreement, this is unlikely to be a major source of bias. Finally, under-ascertainment of healthcare use in administrative records (e.g., due to imperfect data linkage) would attenuate our estimates of agreement with self-reported healthcare use. While these specific limitations relate to this study, similar limitations apply to many record linkage studies, and warrant consideration when deciding on the appropriate methodology for research.

## Conclusion

In contrast to suggestions that self-reported healthcare use in marginalised populations is unreliable (Reijneveld and Stronks 2001, Bhandari and Wagner 2006), we found that in a large cohort of adults recently released from prison, there was reasonably good agreement between self-report and administrative records for use of primary care, emergency department and hospital services, and for use of prescribed medications. Agreement was especially good for GP contact, antidepressant and antipsychotic medication use, and over shorter recall periods. Administrative healthcare records have limitations and are not an unambiguous gold standard; self-report may be a valid and reliable way of collecting information on healthcare use in vulnerable populations.
